# Opioid and benzodiazepine requirements in neonatal and pediatric patients undergoing venoarterial ECMO: insights from a predominantly cardiac cohort

**DOI:** 10.3389/fped.2026.1825420

**Published:** 2026-07-21

**Authors:** Somayah A. Alsuhaibani, Ashjan F. Alghanem, Ibrahim A. Alredaini, Shaimaa Alsulami, Luluah A. AlTukhaifi, Reem M. Beheri, Mashael AlFaifi, Renad A. Alshuraim, Lama Alfehaid, Khalid Al Sulaiman

**Affiliations:** 1 Pharmaceutical Care Division, King Faisal Specialist Hospital & Research Centre, Riyadh, Saudi Arabia; 2 Pharmaceutical Care Division, Maternity and Children's Hospital, Qassim Health Cluster, Buraidah, Saudi Arabia; 3 Clinical Research Department, King Faisal Specialist Hospital & Research Centre, Riyadh, Saudi Arabia; 4 Department of Pharmacy Practice, College of Pharmacy, Umm Al-Qura University, Makkah, Saudi Arabia; 5 Critical Care Medicine Department, King Faisal Specialist Hospital & Research Centre, Riyadh, Saudi Arabia; 6 Department of Pharmaceutical Care Services, King Saud Medical City, Riyadh, Saudi Arabia; 7 Department of Clinical Trial, Research and Innovation Center, Riyadh, Saudi Arabia; 8 Pharmacy Practice Department, College of Pharmacy, King Saud bin Abdulaziz University for Health Sciences, Riyadh, Saudi Arabia; 9 Department of Pharmaceutical Care Services, King Abdulaziz Medical City, Ministry of National Guard-Health Affairs, Riyadh, Saudi Arabia; 10 King Abdullah International Medical Research Center (KAIMRC), Ministry of National Guard Health Affairs (MNGHA), Riyadh, Saudi Arabia; 11 Saudi Critical Care Pharmacy Research (SCAPE) Platform, Riyadh, Saudi Arabia; 12 Saudi Society for Multidisciplinary Research Development and Education (SCAPE Society), Riyadh, Saudi Arabia

**Keywords:** analgesia, benzodiazepines, critical care, ECMO, opioids, pediatrics, sedation

## Abstract

**Background:**

Managing sedation and analgesia in neonates and pediatric patients supported with extracorporeal membrane oxygenation (ECMO) is challenging due to altered pharmacokinetics, prolonged critical illness, and the need for deep sedation to maintain circuit integrity. Limited data describing cumulative opioid and benzodiazepine exposure and factors associated with dose escalation in pediatric ECMO populations.

**Methods:**

We conducted a retrospective cohort study of neonates and pediatric patients aged ≤14 years who received ≥72 h of venoarterial ECMO support in a pediatric cardiac surgical intensive care unit between 2019 and 2023. Exclusions included early mortality, neurological conditions requiring altered sedation strategies, or sedation primarily used for seizure management. Opioid and benzodiazepine doses administered during ECMO were converted to intravenous morphine and midazolam equivalents. Cumulative (mg/kg) and average daily (mg/kg/day) exposures were calculated. Multivariable regression analyses were performed to identify clinical predictors of sedative and analgesic dose requirements and associations with outcomes.

**Results:**

Eighty patients were included, with postoperative cardiac disease accounting for 68.8% of primary diagnoses. All patients received opioids, and 96.3% received benzodiazepines; 66.3% received adjunctive sedative agents. Median cumulative opioid and benzodiazepine exposures were 7.33 mg/kg and 15.75 mg/kg, respectively. Prolonged ICU length of stay and ECMO duration were independently associated with higher cumulative opioid exposure (Coeff = 0.02; 95% CI: 0.01 to 0.03; *p* = 0.001 and Coeff = 0.02; 95% CI: 0.01 to 0.04; *p* = 0.001, respectively). Furthermore, multivariable analysis demonstrated that higher in-hospital mortality was significantly associated with prolonged ICU length of stay (Coeff = 0.73; 95% CI: 0.12 to 1.34; *p* = 0.02), higher average opioid doses (Coeff = 1.75; 95% CI: 0.11 to 3.39; *p* = 0.04), acute kidney injury (Coeff = 3.34; 95% CI: 0.32 to 6.35; *p* = 0.03), and longer ECMO duration (Coeff = 0.43; 95% CI: 0.06 to 0.80; *p* = 0.02).

**Conclusion:**

Prolonged ICU stays and ECMO courses in neonatal and pediatric patients significantly increase cumulative opioid burdens. Crucially, higher average opioid doses, acute kidney injury, and extended ICU and ECMO durations serve as key clinical predictors of increased in-hospital mortality. These findings highlight the need for individualized, physiology-informed sedation strategies and structured weaning approaches to optimize outcomes in this high-risk population.

## Introduction

Sedation and analgesia are critical elements of care for neonates and pediatric patients undergoing invasive procedures, mechanical ventilation, and other distressing interventions in the intensive care unit. Thus, clinicians must balance adequate comfort, ventilator synchrony, and procedural safety against the risks of oversedation, drug accumulation, tolerance, delirium, and iatrogenic withdrawal. These challenges are exacerbated by developmental pharmacokinetic variability, organ dysfunction, critical illness severity, and the need for individualized sedation targets (1).

Extracorporeal membrane oxygenation (ECMO) is a form of cardiopulmonary bypass for patients with respiratory, cardiac, or combined cardiopulmonary failure unresponsive to conventional treatments (25). Due to its invasive nature and potential for serious complications, ECMO patients typically require deep sedation to prevent accidental decannulation and ensure optimal positioning to support ECMO flows. In critically ill patients, the conventional approach involves using analgesics and sedatives during mechanical ventilation to mitigate physiological responses and reduce morbidity and mortality (3). Opioids and benzodiazepines remain widely used for analgesia and sedation in critically ill patients, particularly during mechanical ventilation and ECMO support; however, current practice increasingly incorporates adjunctive agents such as dexmedetomidine, ketamine, and propofol to achieve targeted sedation while limiting cumulative opioid and benzodiazepine exposure (2).

Sedation and analgesia during ECMO present unique challenges. Patients supported with ECMO often require higher cumulative exposure of sedatives and analgesia due to ECMO-related pharmacokinetic changes, circuit drug sequestration, the need to prevent agitation-related mechanical complications prolonged mechanical ventilation, and longer intensive care unit (ICU) courses that may promote tolerance and delay sedation weaning (4, 5). Franciscovich et al. evaluated sedation in 87 neonates receiving ECMO and found that opioid and sedative exposure increased over time, despite no consistent correlation between pain scores and medication administration. This suggests that dose escalation may have reflected ECMO-related sedation goals, clinical instability, or prevention of agitation-related complications rather than pain severity alone (6). Anton-Martin et al. analyzed 160 pediatric and neonatal patients and found that prolonged ECMO runs were associated with higher sedation requirements, with significant reliance on opiates and benzodiazepines (17). In addition, another study investigated drug adsorption in ECMO circuits, highlighting that lipophilic drugs like fentanyl and midazolam are highly sequestered, whereas morphine exhibited minimal sequestration (7). Lastly, Schneider et al. (8), in a secondary analysis of the RESTORE trial, found that ECMO patients required higher doses of opioids and benzodiazepines, leading to more frequent iatrogenic withdrawal symptoms compared to non-ECMO patients. Notably, a goal-directed sedation strategy (RESTORE protocol) resulted in lower opioid exposure and shorter sedation duration than usual care (8). Sedation and analgesia management in pediatric ECMO patients is influenced by several factors, such as drug exposure, age, ICU length of stay (LOS) and mechanical ventilation duration, all of which contribute to the risk of iatrogenic withdrawal.

While multiple studies have examined sedative and analgesic requirements in adult ECMO patients, there is limited data on neonates and pediatric ECMO patients. Therefore, this study aims to evaluate opioid and benzodiazepine dose requirements in neonates and pediatric patients with ECMO support and identify factors associated with medication dose escalation.

## Method

### Study design

We conducted a retrospective cohort study of critically ill neonates and pediatric patients admitted to the Cardiac Surgical Intensive Care Unit for Pediatrics (CSICU-P) from January 1, 2019, to December 31, 2023. Eligible patients included those who received ECMO support and were administered sedative and analgesic medications during their ECMO course. For these patients, opioid doses were standardized to Intravenous (IV) morphine equivalents and benzodiazepine doses to IV midazolam equivalents. The study protocol was reviewed and approved by the hospital Institutional Review Board (RAC#2241124).

### Study subjects

**Figure 1:**
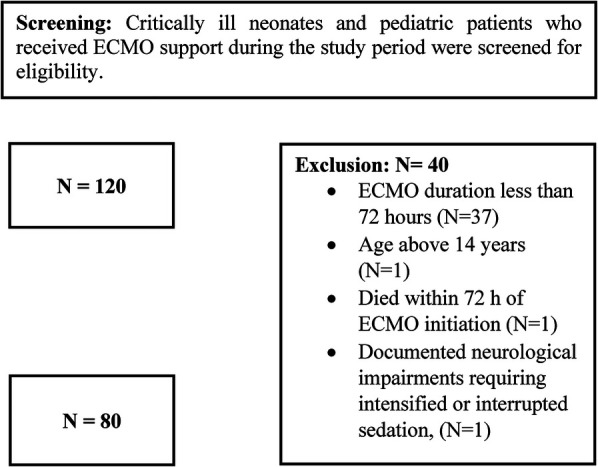
Flowchart for eligibility criteria.

Critically ill neonates and pediatric patients aged 14 years or younger who received ECMO support during the study period were screened for eligibility. Patients were excluded if they received ECMO support for less than 72 h, did not receive sedative and/or analgesic medications during ECMO, died within 72 h of ECMO initiation, had documented neurological impairments requiring intensified or interrupted sedation, or if sedatives were used for managing status epilepticus (Figure 1) illustrates the study flow charts.

### Study setting

This study was conducted at King Faisal Specialist Hospital and Research Centre (KFSH&RC), Riyadh, Saudi Arabia, a government-owned, non-profit tertiary and quaternary academic medical center accredited by the Joint Commission International and affiliated with Alfaisal University. The Riyadh campus has 1,500 beds and serves as a national referral hub for specialized care, including transplantation, cardiology, oncology, and pediatric subspecialties. Pediatric cardiac surgical patients are managed in a dedicated 20-bed CSICU-P, operated as a closed unit led by pediatric intensivists in collaboration with cardiac surgeons, anesthesiologists, and cardiologists. ECMO support was delivered according to institutional protocols using standard circuit configurations that are typically based on centrifugal pump systems. However, specific technical details, including pump type and circuit components, were not consistently documented in the medical records and were therefore not included in the analysis.

### Study outcomes

This study aims to evaluate opioid and benzodiazepine dose requirements in neonates and pediatric patients on ECMO support. The primary outcome is to estimate the average and cumulative dose requirements of opioids and benzodiazepines in this population. Secondary outcomes include assessing factors associated with higher sedation and analgesia dose requirements—such as age, post-operative pulmonary hypertension, and the number of procedures—as well as clinical outcomes, including ventilation days, ICU length of stay, mortality, and Do Not Resuscitate (DNR) status.

The established cutoff limits for high doses of opioids and benzodiazepines are based on the average and cumulative doses documented in studies conducted within pediatric intensive care units (8).

### Outcomes definition

- **Morphine Equivalent Dose:** Opioid exposure was determined by converting hourly doses of fentanyl and hydromorphone to their equivalent intravenous (IV) morphine doses using established equianalgesic conversion ratios (9). IV morphine did not require conversion. This standardization enables uniform comparison of opioid administration across different agents.
- **Midazolam Equivalent Dose:** The only benzodiazepine agent administered to patients in this study was IV midazolam. No dose conversions or midazolam-equivalent calculations were necessary.
- **Hepatic dysfunction:** Defined as an elevation of alanine aminotransferase (ALT) and/or aspartate aminotransferase (AST) greater than three times the upper limit of normal (10).
- **Acute kidney injury (AKI):** Classified according to the Pediatric Risk, Injury, Failure, Loss, End Stage Renal Disease (pRIFLE criteria) (Risk: estimated creatinine clearance (eCCL) decrease ≥25%; Injury: eCCL decrease ≥50%; Failure: eCCL decrease ≥75%; Loss: renal failure >4 weeks; End-stage: failure >3 months) and the Acute Kidney Injury Network (AKIN) criteria for neonates (Stage I: serum creatinine increase >0.3 mg/dL or 150%–200% from baseline; Stage II: 200%–300%; Stage III: >300% or absolute Scr ≥2.5 mg/dL, or dialysis requirement) (11, 12).

### Data collection

Data were extracted from EHR and systematically entered into a REDCap-based data collection form developed specifically for this study. Information was gathered from the initiation of ECMO support through to decannulation or death, whichever occurred first. The variables collected encompassed demographic characteristics at the initiation of ECMO (including date of birth, age, gender, weight, and height), as well as the primary diagnosis. Diagnoses were classified into categories such as neonatal lung disease, pediatric respiratory failure, non-surgical cardiac disease, postoperative cardiac condition, septic shock, or other specified conditions. Relevant clinical data included the indication for ECMO support [pulmonary failure, cardiac failure, or extracorporeal cardiopulmonary resuscitation (ECPR)], ECMO type [venovenous (VV) or venoarterial (VA)], and cannulation site (thoracic, femoral-internal jugular, or femoral). The duration of ECMO support (in days) was documented, along with the filtration method utilized where applicable (ultrafiltration, hemodiafiltration, or hemofiltration).

Opioid doses were documented and standardized by converting all doses to IV morphine equivalents; no conversion was necessary if IV morphine was administered (9). For benzodiazepines, midazolam was the only agent used in the study, and no dose conversions or midazolam-equivalent calculations were required. Cumulative doses (mg/kg over the total ECMO duration) and average daily doses (mg/kg/day) doses of opioids and benzodiazepines administered during ECMO were calculated. Adjunctive sedative medications—including dexmedetomidine, ketamine, and propofol—were also recorded. The use of muscle relaxants and other medications with potential sedative or neurologic effects (e.g., caffeine citrate, phenobarbital, diphenhydramine, erythromycin, and clarithromycin) was also captured. Factors potentially influencing sedation requirements—such as pulmonary hypertension (as documented in the electronic health records (EHR)), the number of procedures performed during ECMO (including any procedures involving a skin incision, such as chest tube placement or cannula revision, but excluding cannulation and decannulation), and the presence of organ dysfunction (renal or hepatic)—were also documented.

Sedation depth and pain were assessed using validated pediatric tools, including the COMFORT-Behavior (COMFORT-B) scale and the Face, Legs, Activity, Cry, Consolability (FLACC) score. Sedation scores were recorded every hour throughout the ECMO course, and daily average scores were calculated for each patient. Sedation depth was classified as adequate, deeply sedated, or agitated, with adequate sedation defined as a COMFORT-B score between 11 and 23 (13). Pain was assessed using the FLACC score, where a score of 0 indicates no pain, and a score >4 indicates persisting pain (14). Iatrogenic withdrawal symptoms were evaluated using the Withdrawal Assessment Tool-1 (WAT-1), recorded during the first 48 h following sedation weaning (15). Additional data included the use and duration of intermittent sedative medications used to facilitate weaning.

### Statistical analysis

Descriptive statistics were used to summarize baseline demographic and clinical variables. Categorical variables were reported as frequencies and percentages, while continuous variables were presented as medians and interquartile ranges (IQRs) due to non-normal data distributions. Comparisons of cumulative and average daily sedative exposure across primary diagnosis categories were performed using the Kruskal–Wallis test, given the non-parametric distribution of dose variables. For dichotomous clinical outcomes—including mortality, development of AKI, hepatic dysfunction, DNR status, pulmonary hypertension, and use of adjuvant sedative medications—group differences in sedative exposure were assessed using the Mann–Whitney *U*-test.

Multivariable linear regression models were constructed to identify predictors of cumulative and average daily opioid and benzodiazepine exposure. A stepwise regression analysis was additionally conducted to identify statistically retained predictors. A backward elimination approach was applied, in which all clinically relevant variables were initially included in the full model and sequentially removed using a removal criterion of *p* > 0.10. For linear regression models, model assumptions and goodness-of-fit were assessed using adjusted *R*^2^ and root mean square error (RMSE). Logistic regression was applied where appropriate, with caution given to evidence of complete separation in small outcome subgroups. For the logistic regression model, model fit was evaluated using the Hosmer–Lemeshow goodness-of-fit test and the area under the receiver operating characteristic curve (AUC). All analyses were performed using STATA version 18, and a two-sided *p*-value < 0.05 was considered statistically significant.

## Results

A total of 120 patients who underwent ECMO were screened, with 80 included in the study (Figure 1). Cardiac failure accounted for 75% of ECMO indications, with cardiac arrest coming in second at 18.8% followed by respiratory failure at 6.2%. Among the patients included, 100% were on VA ECMO. The median duration of ECMO was 6 days (IQR: 6). Most patients were cannulated via thoracic cannulation (98.8%), with only one patient needing femoral cannulation.

### Demographic and clinical characteristics

The gender distribution was balanced, with 50% male patients. The median body weight at the start of ECMO was 3.5 kg (IQR: 4.7), while the median age of the participants was 3 months (IQR: 8). Post-surgical cardiac complications were the most common primary ICU diagnosis (68.8%), followed by non-surgical cardiac disease (8.8%), septic shock (3.8%), respiratory failure (2.5%), and Neonatal lung disease (1.3%) (Table 1).

**Table 1:** Patients’ demographics and clinical characteristics (*n* = 80).

Variable	Overall (*n* = 80)
Patient Demographics	
Gender (male), *n*(%)	40 (50.0)
Age at starting ECMO (months), **median [IQR]**	3 [8]
Weight (kg), **median [IQR]**	3.5 [4.7]
Primary diagnosis, *n*(%)	
Postoperative cardiac	55 (68.8)
Non-surgical cardiac	7 (8.8)
Septic shock	3 (3.8)
Pediatric respiratory failure	2 (2.5)
Neonatal lung disease	1 (1.3)
Other, (%)	12 (15.0)
ECMO indication, *n*(%)	
Pulmonary Failure	5 (6.3)
Cardiac Failure	60 (75.0)
Extracorporeal Cardiopulmonary Resuscitation (ECPR)	15 (18.8)
Cannulation site, *n*(%)	
Thoracic (central)	79 (98.8)
Femoral (peripheral)	1 (1.3)
Type of ECMO, *n*(%)	
VA-ECMO	80 (100)
Type of ECMO filtration used, *n*(%)	
Hemodiafiltration	11 (13.8)
Ultrafiltration	10 (12.5)
Hemofiltration	26 (32.5)
None	33 (41.25)
Sedative medications used during ECMO, *n*(%)	
Opioid	80 (100.0)
– Morphine	76 (95.0)
– Fentanyl	60 (75.0)
– Hydromorphone	2 (2.5)
Benzodiazepine	77 (96.3)
– Midazolam	77 (96.3)
Adjunctive sedation	53 (66.3)
– Ketamine	43 (53.8)
– Propofol	1 (1.3)
– Dexmedetomidine	8 (10.0)
– Muscle relaxant	32 (40.0)
Other medications used during ECMO, *n*(%)	
Phenobarbital	1 (1.3)
Diphenhydramine	2 (2.5)
ECMO related variables	
Duration of ECMO (days), **median [IQR]**	6 [6]
Duration of sedation during ECMO support (days), **median [IQR]**	6 [6]
Iatrogenic Withdrawal symptoms in first 48 h, *n*(%)	
Yes, (%)	4 (5.0)
No, (%)	12 (15.0)
Not reported, (%)	38 (47.5)
Patient death, (%)	26 (32.5)
Procedures During ECMO	
Number of procedures done during ECMO, **median [IQR]**	2 [3]
Organ dysfunction, support, and complications during ECMO	
Presence of pulmonary hypertension during ECMO (%)	18 (22.5)
Accidental decannulation, *n*(%)	7 (8.8)
Mechanical ventilation	
Duration of mechanical ventilation from the start of ECMO (days)	13 [13]
Reintubation rate, *n*(%)	18 (22.8)
Duration of mechanical ventilation after reintubation (days), **median [IQR]**	6 [15]
Renal dysfunction during ECMO	
Development of AKI during ECMO, *n*(%)	22 (27.5)
Stage of AKI (pRIFLE for pediatric), *n*(%)	**15** **(18.8)**
– Risk	7 (46.7)
– Injury	5 (33.3)
– Failure	1 (6.7)
– End-stage	2 (13.3)
Stage of AKI (AKIN for neonates), *n*(%)	**7** **(8.8)**
– AKI Stage 1	5 (71.4)
– AKI Stage 2	1 (14.3)
– AKI Stage 3	1 (14.3)
Renal replacement therapy during ECMO, *n*(%)	
Peritoneal dialysis (PD)	16 (43.2)
Hemodialysis (HD)	18 (48.6)
Continuous renal replacement therapy (CRRT)	3 (8.1)
Liver dysfunction during ECMO	
Development of hepatic dysfunction during ECMO, (%)	49 (61.3)
Highest AST level during ECMO, median [IQR]	281 [919.8]
Highest ALT level during ECMO, median [IQR]	224.5 [165]
Overall outcome	
Hospital length of stay (days), median [IQR]	35 [42.5]
ICU length of stay (days), median [IQR]	29 [36.5]
Mortality rate, (%)	40 (50.0)
DNR code, (%)	4 (5.0)

ECMO, extracorporeal membrane oxygenation; SBS, state behavioral scale; FLACC: AKI, acute kidney injury; WAT, withdrawal assessment tool; AKIN; AST; ALT; ICU, intensive care unit; DNR, do not resuscitate.

### Organ (s) dysfunction and clinical outcomes

Acute Kidney Injury (AKI) developed in twenty-two patients (27.5%), fifteen patients (18.8%) were pediatric, and seven patients (8.8%) were neonates. Using the pRIFLE staging scale for AKI, 46.7% of patients were at the Risk stage, and 33.3% of patients at the injury stage. In contrast, using the AKIN staging scale for neonates, the majority of patients were in stage 1 (71.4%). A total of 37 patients required renal replacement therapy (RRT) during ECMO. The most common type of RRT utilized was Hemodialysis (HD) (48.6%), followed by Peritoneal dialysis (PD) (43.2%) and Continuous renal replacement therapy (CRRT) (8.1%).

During ECMO support, 49 patients (61%) experienced hepatic dysfunction, with a median peak ALT level of 224.5 (IQR: 165) and maximum AST levels reaching 281 (IQR: 919.8). Additionally, 18 patients (22.5%) presented with pulmonary hypertension while on ECMO. The median duration of mechanical ventilation after ECMO initiation was 13 days (IQR: 13). Eighteen patients required reintubation, with a median duration of mechanical ventilation of 6 days following reintubation. Furthermore, 5% of patients experienced iatrogenic withdrawal symptoms within the initial 48 h of sedation weaning, and 8.8% had incidents of accidental decannulation. The median number of procedures performed during ECMO was 2 (IQR: 3), as outlined in Table 1.

LOS in the ICU for patients on ECMO was 29 days (IQR 36.5). In contrast, the overall hospital LOS averaged 35 days (IQR: 42.5). Among the patients, four (5%) were designated DNR, and the overall mortality rate was 50%, as indicated in Table 1.

### Analgesic and sedative exposure

Opioids were administered to all patients, most commonly morphine (95%), followed by fentanyl (75%), and hydromorphone (2.5%). The median Comfort-B scale score was 12 (IQR 2), and approximately 36% of patients reported experiencing no pain, as indicated by the FLACC score. Benzodiazepine was prescribed to 96.3% of the patients, specifically midazolam, with a median duration of six days while on ECMO. Almost 66.3% of the total population received at least one adjuvant sedative drug. The most commonly used were ketamine (53.8%), dexmedetomidine (10%), and propofol (1.3%). Additionally, neuromuscular blockers were given in 40% of cases, as shown in Table 1.

In Table 2, the data indicates that the median cumulative opioid dose was 7.33 mg/kg (IQR 8.72), whereas the cumulative dose of benzodiazepines was 15.75 mg/kg (IQR 24.9). The average daily doses for opioids and benzodiazepines were 0.71 mg/kg/day and 0.20 mg/kg/day, respectively.

**Table 2:** Analgesic and sedative medication doses and pain scales.

Cumulative and average daily dose	Overall (*n* = 80)
Opioid, cumulative dose (mg/kg), **median [IQR]**	7.33 (8.72)
Opioid, average daily dose (mg/kg/day), median [IQR]	0.708 (0.0425)
Benzodiazepine, cumulative dose (mg/kg), median [IQR]	15.75 (24.9)
Benzodiazepine, average daily dose (mg/kg/day), median [IQR]	0.195 (0.178)
Pain, sedation, and comfort measures	
Comfort-B scale, **median [IQR]**	12 [2]
FLACC score, average daily, *n*(%)	
– No pain	29 (36.3)

### Factors associated with increased cumulative doses of opioids

The multiple linear regression model aimed to identify clinical predictors of cumulative opioid exposure, using a log-transformed outcome variable to account for skewness in dosing distribution (Table 3). The model was statistically significant [F(4, 75) = 7.45, *p* < 0.0001], with an R² of 0.2843 and an adjusted *R*^2^ of 0.2461, indicating that approximately one-quarter of the variance in cumulative opioid dose was explained by the included predictors. The model included both continuous measures of clinical exposure (hospital stay, ICU stay, ECMO duration) and a binary procedural complication (accidental decannulation), offering a multidimensional view of factors influencing opioid administration. The findings suggest that opioid dosing in critical care settings is shaped by both the intensity and duration of medical interventions. Prolonged ICU and ECMO support were associated with increased cumulative opioid doses (Coeff = 0.02; 95% CI: 0.01 to 0.03; *p* = 0.001 and Coeff = 0.02; 95% CI: 0.01 to 0.04; *p* = 0.001, respectively) (Table 3). There was a marginal association between lower cumulative opioid doses and accidental decannulation (Coeff = −0.39; 95% CI: −0.82 to 0.05; *p* = 0.08).

**Table 3:** Multiple linear regression model for predicting cumulative opioid dose (mg/kg).

Variable	Coefficient (B)	Std. Error	z/t	*p*-value	95% CI
Hospital length of stay	−0.0169	0.0054	−3.10	0.003	[−0.0277, −0.0060]
ICU length of stay	0.0202	0.0056	3.61	0.001	[0.0090, 0.0313]
ECMO duration	0.0240	0.0071	3.36	0.001	[0.0098, 0.0382]
Accidental decannulation	−0.3887	0.2177	−1.79	0.078	[−0.8223, 0.0450]

### Factors associated with increased opioid average doses

A multiple linear regression analysis was conducted to examine the relationship between average opioid dose (log-transformed) and selected clinical predictors, including age at ECMO initiation, mechanical ventilation status, and weight at ECMO start (Table 4). The model was statistically significant [F(3, 65) = 33.24, *p* < 0.0001], indicating that the included variables collectively explain a substantial proportion of the variance in opioid dosing. The model yielded an *R*^2^ of 0.6054 and an adjusted *R*^2^ of 0.5872, suggesting that approximately 59%–61% of the variability in average opioid dose was accounted for by the predictors. Multivariable analysis demonstrated that an increase in age at ECMO initiation and mechanical ventilation were significantly associated with a *lower* average daily opioid dose (Coeff = −0.06; 95% CI: −0.09 to −0.03; *p* = 0.001 and Coeff = −0.04; 95% CI: −0.07 to −0.00; *p* = 0.03, respectively). Conversely, patient weight at ECMO initiation was positively associated with a *higher* average opioid dose (Coeff = 0.06; 95% CI: 0.03 to 0.10; *p* < 0.001).

**Table 4:** Multiple linear regression model for predicting average daily opioid dose (mg/kg/day).

Variable	Coefficient (B)	Std. error	z/t	*p*-value	95% CI
Age at ECMO start	−0.0613	0.0168	−3.64	0.001	[−0.0949, −0.0277]
Mechanical ventilation	−0.0352	0.0162	−2.17	0.034	[−0.0676, −0.0027]
Weight at ECMO start	0.0629	0.0167	3.77	<0.001	[0.0296, 0.0962]

### Factors associated with increased benzodiazepine cumulative doses

A multiple linear regression analysis was conducted to identify predictors of cumulative benzodiazepine exposure (log-transformed) among patients undergoing ECMO (Table 5). The final model, selected using a stepwise procedure with entry and removal thresholds of *p* < 0.05 and *p* ≥ 0.10 respectively, retained five predictors and demonstrated a statistically significant overall fit [F(5, 66) = 8.45, *p* < 0.0001]. The model explained approximately 39% of the variance in cumulative benzodiazepine dose (*R*^2^ = 0.3902; adjusted *R*^2^ = 0.3440), indicating moderate explanatory power. Three continuous clinical variables were significantly associated with benzodiazepine exposure. A lower weight at the initiation of ECMO was significantly associated with higher cumulative benzodiazepine doses (Coeff = −0.06; 95% CI: −0.09 to −0.03; *p* = 0.001). Longer ECMO duration was significantly associated with lower cumulative benzodiazepine doses (Coeff = −0.04; 95% CI: −0.07 to −0.03; *p* = 0.03). Regarding diagnostic categories, patients with non-surgical cardiac diagnoses and those with postoperative cardiac diagnoses received significantly higher cumulative benzodiazepine doses compared to the reference group (Coeff = 1.74; 95% CI: 0.59 to 2.89; *p* = 0.003 and Coeff = 1.22; 95% CI: 0.29 to 2.16; *p* = 0.01, respectively).

**Table 5:** Multiple linear regression model for predicting cumulative benzodiazepine dose (mg/kg).

Variable	Coefficient (B)	Std. Error	z/t	*p*-value	95% CI
Weight at ECMO start	−0.0613	0.0168	−3.64	0.001	[−0.0949, −0.0277]
ECMO duration	−0.0352	0.0162	−2.17	0.034	[−0.0676, −0.0027]
Sedation duration on ECMO	0.0629	0.0167	3.77	<0.001	[0.0296, 0.0962]
Primary diagnosis: non-surgical cardiac	1.7407	0.5743	3.03	0.003	[0.5940, 2.8873]
Primary diagnosis: postoperative cardiac	1.2241	0.4663	2.63	0.011	[0.2932, 2.1551]

### Factors associated with increased benzodiazepine average dose

A multiple linear regression analysis was performed to identify predictors of average benzodiazepine dose (log-transformed) among patients undergoing ECMO (Table 6). The model included three independent variables: in-hospital mortality, ECMO duration, and weight at ECMO initiation. All variables met the inclusion criteria under a stepwise selection procedure (entry *p* < 0.05, removal *p* ≥ 0.10), and the final model was statistically significant [F(3, 66) = 21.42, *p* < 0.0001]. The model explained approximately 49% of the variance in benzodiazepine dosing (*R*^2^ = 0.4934; adjusted *R*^2^ = 0.4703), indicating moderate explanatory power. The RMSE was 0.8618, reflecting acceptable residual variability. In-hospital mortality and prolonged ECMO duration were significantly associated with lower average benzodiazepine doses (Coeff = −0.061; 95% CI: −0.09 to −0.03; *p* = 0.001 and Coeff = −0.04; 95% CI: −0.06 to −0.003; *p* = 0.04, respectively). In contrast, higher weight at ECMO initiation (Coeff = 0.07; 95% CI: 0.03 to 0.10; *p* < 0.001) was significantly associated with increased average benzodiazepine doses.

**Table 6:** Multiple linear regression model for predicting average daily benzodiazepine dose (mg/kg/day).

Variable	Coefficient (B)	Std. Error	z/t	*p*-value	95% CI
In-hospital mortality	−0.0613	0.0168	−3.64	0.001	[−0.0949, −0.0277]
ECMO duration	−0.0352	0.0162	−2.17	0.034	[−0.0676, −0.0027]
Weight at ECMO start	0.0629	0.0167	3.77	<0.001	[0.0296, 0.0962]

### Factors associated with mortality

A multivariable logistic regression model was developed to identify predictors of in-hospital mortality among 65 patients (Table 7). The model demonstrated strong overall performance, with a likelihood ratio chi-square of 61.13 (df = 7, *p* < 0.0001) and a pseudo R² of 0.6785, indicating that approximately 68% of the variance in mortality outcomes was explained by the included predictors. The independent variables retained in the model met the stepwise selection criteria (entry *p* < 0.05, removal *p* < 0.10). Multivariable analysis demonstrated that higher in-hospital mortality was significantly associated with prolonged ICU length of stay (Coeff = 0.73; 95% CI: 0.12 to 1.34; *p* = 0.02), higher average opioid doses (Coeff = 1.75; 95% CI: 0.11 to 3.39; *p* = 0.04), acute kidney injury (Coeff = 3.34; 95% CI: 0.32 to 6.35; *p* = 0.03), and longer ECMO duration (Coeff = 0.43; 95% CI: 0.06 to 0.80; *p* = 0.02). Conversely, total hospital length of stay was inversely associated with mortality (Coeff = −0.85; 95% CI: −1.51 to −0.19; *p* = 0.01), indicating a potential survival bias or protective effect. Additionally, a borderline protective trend was observed for average benzodiazepine dose (Coeff = −1.71; 95% CI: −3.43 to −0.016; *p* = 0.05), whereas reintubation showed a marginal association with increased mortality (Coeff = 3.46; 95% CI: −0.40 to 7.31; *p* = 0.07).

**Table 7:** Stepwise logistic regression model for predicting in-hospital mortality.

Variable	Coefficient (B)	Std. Error	z/t	*p*-value	95% CI
Hospital length of stay	−0.8471	0.3376	−2.51	0.012	[−1.509, −0.185]
ICU length of stay	0.7287	0.3133	2.33	0.020	[0.115, 1.343]
Benzodiazepine average dose	−1.7066	0.8790	−1.94	0.052	[−3.429, 0.016]
Opioid average dose	1.7514	0.8381	2.09	0.037	[0.109, 3.394]
Reintubation	3.4571	1.9653	1.76	0.079	[−0.395, 7.309]
Kidney injury (AKI)	3.3369	1.5396	2.17	0.030	[0.319, 6.354]
ECMO duration	0.4300	0.1882	2.28	0.022	[0.061, 0.799]

## Discussion

Sedation and analgesia in neonatal and pediatric ECMO patients present challenges, as drug pharmacokinetics show significant variability within this age group and are greatly affected by critical illness and ECMO-related factors. Despite the growing use of ECMO across the Middle East, there is a lack of regional studies that examine actual sedative or analgesic exposure and the clinical factors influencing dose escalation in these patients. Our research aimed to address this gap by exploring the use of opioids and benzodiazepines in a cohort of 80 neonates and pediatric patients supported with VA-ECMO at a high-volume tertiary institution, illustrating considerable variability in dosing attributable to patient characteristics, diagnosis, and ECMO-related factors.

All patients in our cohort received opioids, and nearly all were treated with benzodiazepines, reflecting the standard practice of maintaining deep sedation to prevent accidental decannulation, optimize ECMO flow, and reduce metabolic demand. The median cumulative opioid dose (7.33 mg/kg) and benzodiazepine dose (15.75 mg/kg) are comparable to what has been reported in previous pediatric ECMO studies. In the RESTORE study, Schneider et al. demonstrated substantial and progressively increasing opioid and benzodiazepine requirements in children receiving ECMO, with exposures markedly higher than those in non-ECMO mechanically ventilated patients (8). Anton-Martin et al. also described rising sedative and analgesic needs over time, particularly among postoperative cardiac patients (17), a pattern consistent with our finding that postoperative cardiac status was a significant predictor of greater cumulative benzodiazepine exposure. Although direct numerical comparisons across studies are challenging because of differences in patient populations, clinical indications, and dosing units, the overall magnitude of sedative and analgesic exposure observed in our cohort aligns with dosing patterns reported in other pediatric ECMO populations (16–18).

Our findings also align with existing evidence that ECMO-related pharmacokinetic alterations contribute to higher sedative requirements. Lipophilic and highly protein-bound agents such as fentanyl and midazolam are known to undergo substantial sequestration within the ECMO circuit, reducing the amount of drug available for clinical effect and prompting the need for higher or more frequent dosing (19). Although morphine is less prone to circuit adsorption than more lipophilic opioids, it was still used extensively in our cohort (95%), and the cumulative doses remain clinically high. This likely reflects the combined influence of ECMO-associated changes in drug disposition, the need to maintain stable and deep sedation, and the severity and complexity of the underlying illness rather than sequestration alone.

Our multivariable analysis demonstrated that cumulative opioid exposure is independently associated with ICU LOS, ECMO duration, and indicators of more complex critical illness. Meanwhile, a more extended overall hospital stay is inversely related to cumulative dosing. These findings suggest that opioid escalation reflects the intensity and length of critical illness during ECMO, rather than total hospitalization time. Similar findings have been reported in the RESTORE cohort, where patients on ECMO received significantly higher and more prolonged opioid and benzodiazepine doses compared with non-ECMO mechanically ventilated patients (16). Data from Shekar et al. in adult ECMO patients also describe increased sedative needs as a typical aspect of ECMO physiology (19). Our study further contributes by quantifying how ICU and ECMO durations independently influence cumulative opioid exposure in a pediatric VA-ECMO population.

The observed inverse association between ECMO duration and cumulative benzodiazepine exposure likely reflects progressive sedation de-escalation and transition to alternative strategies as patients stabilize**.** Prior sedation literature describes an initial preference for benzodiazepine-rich regimens to rapidly achieve deep sedation, followed by a transition toward opioid-based or multimodal approaches as patients stabilize (16, 18, 20). The greater benzodiazepine requirements observed in postoperative and non-surgical cardiac patients in our cohort are also consistent with previous reports that cardiac ECMO patients often need more aggressive sedation due to hemodynamic fragility, frequent interventions, and increased risk of agitation-related complications (17, 21).

Furthermore, younger patients required higher weight-adjusted doses of opioids, and those with lower weight at the start of ECMO needed more weight-adjusted benzodiazepines. This finding aligns with established pediatric pharmacokinetic principles and prior ECMO studies (8, 19, 22). Neonates and infants have a larger weight-normalized volume of distribution, reduced plasma protein binding, and immature hepatic metabolic pathways, including limited UGT2B7 activity for morphine metabolism and significantly reduced CYP3A4/5 function, affecting midazolam clearance. These developmental factors decrease effective drug concentrations and delay the attainment of steady-state levels, thereby requiring higher mg/kg doses to achieve appropriate sedation (23–25).

These vulnerabilities are further amplified during ECMO. Small infants are disproportionately impacted by drug sequestration within the circuit, as the priming volume often approaches their total blood volume. While morphine is less lipophilic and therefore less extensively adsorbed compared with fentanyl or midazolam, measurable circuit loss still occurs, and benzodiazepines, especially midazolam, demonstrate marked sequestration, with *ex vivo* studies reporting losses of up to 80%–90% over 24 h (22). The combination of developmental physiology and ECMO-related pharmacokinetic changes reduces drug bioavailability and increases dosing requirements in younger and smaller patients.

These mechanisms align with existing clinical observations. Anton-Martin et al. reported significantly higher opioid and benzodiazepine requirements in younger pediatric ECMO patients, particularly those undergoing postoperative cardiac support (17). Similarly, studies by Zimmerman and Raffaeli highlight that neonates experience the most notable ECMO-related changes in drug disposition, especially for highly protein-bound or lipophilic sedatives (18, 21). Shekar et al. also noted increased sedation requirements across ECMO populations, attributing them to circuit sequestration and the need to maintain deep, stable sedation to prevent agitation and preserve circuit integrity (19).

Concurrently, these developmental and ECMO-specific pharmacokinetic factors provide a physiological explanation for the increased sedative and analgesic requirements observed in younger, lower-weight patients in our cohort. Despite high sedative exposure, most patients achieved Comfort-B scores consistent with adequate or deep sedation, and over one-third had FLACC scores indicating no pain. Early iatrogenic withdrawal symptoms within 48 h of weaning were documented in 5% of patients, though incomplete recording of WAT-1 scores suggests this may underestimate the true incidence. Prior pediatric ECMO and PICU data report higher iatrogenic withdrawal rates, particularly in cohorts with prolonged high-dose opioid and benzodiazepine exposure (26, 27). These findings emphasize the need for structured iatrogenic withdrawal monitoring and standardized tapering protocols in ECMO patients. Importantly, these observations likely reflect underlying illness severity and duration of ECMO support, rather than direct effects of sedative exposure alone.

Our cohort's overall 50% mortality rate aligns with previously published outcomes for pediatric VA-ECMO, especially in high-risk cardiac groups (28–30). In our multivariable analysis, mortality correlated with AKI, extended ECMO duration, and increased average opioid exposure, while reintubation showed a similar pattern. These factors are recognized as indicators of illness severity and reduced physiological reserve in ECMO patients (17, 31). The observed inverse relationship between benzodiazepine exposure and mortality should be interpreted carefully, as it probably indicates shorter exposure times in patients who died early during ECMO rather than any protective sedative effect.

Our findings highlight key considerations for sedation management in neonatal and pediatric VA-ECMO. Lower weight at the initiation of ECMO consistently required higher weight-adjusted doses, supporting the need for age- and size-specific dosing strategies and early adoption of multimodal, opioid-sparing approaches. The strong association between opioid exposure and both ICU and ECMO duration suggests that sedation requirements largely reflect illness severity and clinical course. Tailoring sedation protocols, particularly in postoperative and non-surgical cardiac patients, may improve stability while minimizing drug accumulation.

Our study has several strengths, including a relatively large single-center pediatric VA-ECMO cohort, the standardized conversion of all sedatives into morphine and midazolam equivalents, and the use of multivariable models to identify independent predictors of cumulative drug exposure. Nonetheless, certain limitations must be acknowledged. The retrospective design introduces the potential for unmeasured confounding, and incomplete documentation of sedation and iatrogenic withdrawal scores limits the precision with which these outcomes can be interpreted. Additionally, this was a single-center study, which may affect generalizability, and we did not perform direct drug-level measurements, leaving pharmacokinetic explanations to be inferred from existing literature. Furthermore, details on ECMO pump type and circuit components were inconsistently available and were not included in the analysis.

Future research should focus on prospective evaluation of specific sedative and analgesic strategies, including opioid-sparing regimens, dexmedetomidine-based protocols, and ketamine adjunctive use. Additionally, pharmacokinetic-guided dosing strategies and circuit-specific drug loss assessments are needed to optimize drug selection and dosing in pediatric ECMO populations.

## Conclusion

In this single-center cohort of neonatal and pediatric patients supported by VA-ECMO, we observed substantial variability in opioid and benzodiazepine exposure. This variation was influenced by patient characteristics, underlying cardiac disease, and the severity and duration of both critical illness and ECMO support. Notably, prolonged ICU stays and extended ECMO courses significantly increased cumulative opioid burdens in these young patients. Crucially, higher average opioid doses, acute kidney injury, and extended ICU and ECMO durations served as key clinical predictors of increased in-hospital mortality. These relationships reflect developmental pharmacokinetic differences that are further complicated by ECMO-related factors, highlighting the critical importance of structured monitoring and standardized weaning strategies. Future prospective studies incorporating pharmacokinetic evaluations and protocolized sedation strategies are needed to optimize care in this population.

## Data Availability

The raw data supporting the conclusions of this article will be made available by the authors, without undue reservation.

## References

[1] Barnes S Yaster M Kudchadkar SR. Pediatric sedation management. Pediatr Rev. (2016) 37(5):203–12. 10.1542/pir.2014-0116 27139328 PMC5812731

[2] Egbuta C Mason KP. Current state of analgesia and sedation in the pediatric intensive care unit. J Clin Med. (2021) 10(9):1847. 10.3390/jcm10091847 33922824 PMC8122992

[3] Tobias JD. Sedation and analgesia in the pediatric intensive care unit. Pediatr Ann. (2005) 34(8):636–45. 10.3928/0090-4481-20050801-12 16149752

[4] Buscher H Vaidiyanathan S Al-Soufi S Nguyen DN Breeding J Rycus P. Sedation practice in veno-venous extracorporeal membrane oxygenation: an international survey. ASAIO J. (2013) 59(6):636–41. 10.1097/MAT.0b013e3182a84558 24088903

[5] Wagner D Pasko D Phillips K Waldvogel J Annich G. *In vitro* clearance of dexmedetomidine in extracorporeal membrane oxygenation. Perfusion. (2013) 28(1):40–6. 10.1177/0267659112456894 22891035

[6] Franciscovich CD Monk HM Brodecki D Rogers R Rintoul NE Hedrick HL. Sedation practices of neonates receiving extracorporeal membrane oxygenation. ASAIO J. (2020) 66(5):559–564. 10.1097/MAT.0000000000001043 31425254

[7] Nasr VG Meserve J Pereira LM Faraoni D Brediger S Goobie S. Sedative and analgesic drug sequestration after a single bolus injection in an ex vivo extracorporeal membrane oxygenation infant circuit. ASAIO J. (2019) 65(2):187–91. 10.1097/MAT.0000000000000793 29595531

[8] Schneider JB Sweberg T Asaro LA Kirby A Wypij D Thiagarajan RR. Sedation management in children supported on extracorporeal membrane oxygenation for acute respiratory failure. Crit Care Med. (2017) 45(10):e1001–10. 10.1097/CCM.0000000000002540 28614197 PMC5600656

[9] Bhatnagar M Pruskowski J. Opioid equivalency. In: StatPearls. Treasure Island (FL): StatPearls Publishing (2025). Available online at: https://www.ncbi.nlm.nih.gov/books/NBK535402/ (Accessed February 29, 2024). 30571023

[10] Patel ST Rajadhyaksha GC Junare P Contractor QQ Souza R Rathi PM. Hepatic dysfunction in medical intensive care unit patients predicts poor outcome. Arq Gastroenterol. (2022) 59(2):164–9. 10.1590/S0004-2803.202202000-31 35830023

[11] Andreoli SP. Acute kidney injury in children. Pediatr Nephrol. (2009) 24(2):253–63. 10.1007/s00467-008-1074-9 19083019 PMC2756346

[12] Askenazi DJ Ambalavanan N Goldstein SL. Acute kidney injury in critically ill newborns: what do we know? What do we need to learn? Pediatr Nephrol. (2009) 24(2):265–74. 10.1007/s00467-008-1060-2 19082634 PMC2755786

[13] Ista E van Dijk M Tibboel D de Hoog M. Assessment of sedation levels in pediatric intensive care patients can be improved by using the COMFORT “behavior” scale. Pediatr Crit Care Med. (2005) 6(1):58–63. 10.1097/01.PCC.0000149318.40279.1A 15636661

[14] Merkel SI Voepel-Lewis T Shayevitz JR Malviya S. The FLACC: a behavioral scale for scoring postoperative pain in young children. Pediatr Nurs. (1997) 23(3):293–7. 9220806

[15] McAlister S Connor JA Engstrand S McLellan MC. Validation of the withdrawal assessment tool-1 (WAT-1) in pediatric cardiovascular patients on an inpatient unit. J Spec Pediatr Nurs. (2023) 28(2):e12404. 10.1111/jspn.12404 36808815

[16] Alhumaid S Alnaim AA Al Ghamdi MA Alahmari AA Alabdulqader M Al Hajji Mohammed SM. International treatment outcomes of neonates on extracorporeal membrane oxygenation (ECMO) with persistent pulmonary hypertension of the newborn (PPHN): a systematic review. J Cardiothorac Surg. (2024) 19(1):493. 10.1186/s13019-024-03011-3 39182148 PMC11344431

[17] Anton-Martin P Modem V Taylor D Potter D Darnell-Bowens C. A retrospective study of sedation and analgesic requirements of pediatric patients on extracorporeal membrane oxygenation (ECMO) from a single-center experience. Perfusion. (2017) 32(3):183–91. 10.1177/0267659116670483 27729502

[18] Zimmerman KO Dallefeld SH Hornik CP Watt KM. Sedative and analgesic pharmacokinetics during pediatric ECMO. J Pediatr Pharmacol Ther. (2020) 25(8):675–88. 10.5863/1551-6776-25.8.675 33214778 PMC7671016

[19] Shekar K Roberts JA Mcdonald CI Fisquet S Barnett AG Mullany DV. Sequestration of drugs in the circuit may lead to therapeutic failure during extracorporeal membrane oxygenation. Crit Care. (2012) 16(5):R194. 10.1186/cc11679 23068416 PMC3682296

[20] Nasr VG DiNardo JA. Sedation and analgesia in pediatric cardiac critical care. Pediatr Crit Care Med. (2016) 17(8 Suppl 1):S225–31. 10.1097/PCC.0000000000000756 27490604

[21] Raffaeli G Pokorna P Allegaert K Mosca F Cavallaro G Wildschut ED. Drug disposition and pharmacotherapy in neonatal ECMO: from fragmented data to integrated knowledge. Front Pediatr. (2019) 7:360. 10.3389/fped.2019.00360 31552205 PMC6733981

[22] Kearns GL Abdel-Rahman SM Alander SW Blowey DL Leeder JS Kauffman RE. Developmental pharmacology—drug disposition in infants and children. N Engl J Med. (2003) 349(12):1157–67. 10.1056/NEJMra035092 13679531

[23] Pacifici GM. Metabolism and pharmacokinetics of morphine in neonates: a review. Clinics (Sao Paulo). (2016) 71:474–80. 10.6061/clinics/2016(08)11 27626479 PMC4975791

[24] de Wildt SN. Profound changes in drug metabolism enzymes and possible effects on drug therapy in neonates and children. Expert Opin Drug Metab Toxicol. (2011) 7(8):935–48. 10.1517/17425255.2011.577739 21548840

[25] Wildschut ED Hanekamp MN Vet NJ Houmes RJ Mathot RA Tibboel D. Feasibility of sedation and analgesia interruption following cannulation in neonates on extracorporeal membrane oxygenation. Intensive Care Med. (2010) 36(9):1587–91. 10.1007/s00134-010-1931-4 20508914 PMC2921052

[26] Ista E van Dijk M Gamel C Tibboel D de Hoog M. Withdrawal symptoms in children after long-term administration of sedatives and/or analgesics: a literature review. “assessment remains troublesome”. Intensive Care Med. (2007) 33(8):1396–406. 10.1007/s00134-007-0696-x 17541548

[27] Jin Y Feng Z Zhao J Hu J Tong Y Guo S. Outcomes and factors associated with early mortality in pediatric postcardiotomy venoarterial extracorporeal membrane oxygenation. Artif Organs. (2021) 45(1):6–14. 10.1111/aor.13773 32645759

[28] Zhou J Wang H Zhao Y Shao J Jiang M Yue S. Short-term mortality among pediatric patients with heart diseases undergoing venoarterial extracorporeal membrane oxygenation: a systematic review and meta-analysis. J Am Heart Assoc. (2023) 12(24):e029571. 10.1161/JAHA.123.029571 38063152 PMC10863771

[29] Singh SP Sengupta S Sahu MK Talwar S Devagourou V Rajashekar P. Outcome of cardiac ECMO in children less than 1 year age undergoing corrective cardiac surgery. J Card Crit Care. (2020) 4(1):25–32. 10.1055/s-0040-1713547

[30] Fleming GM Sahay R Zappitelli M King E Askenazi DJ Bridges BC. The incidence of acute kidney injury and its effect on neonatal and pediatric extracorporeal membrane oxygenation outcomes: a multicenter report from the kidney intervention during extracorporeal membrane oxygenation study group. Pediatr Crit Care Med. (2016) 17(12):1157–69. 10.1097/PCC.0000000000000970 27755398 PMC5138084

[31] Liao MT Tsai IJ Lin FH Tseng LJ Huang SC Chen YS. Risk factors for in-hospital mortality and acute kidney injury in neonatal-pediatric patients receiving extracorporeal membrane oxygenation. J Formos Med Assoc. (2021) 120(9):1758–67. 10.1016/j.jfma.2021.03.004 33810928

